# Diagnosis and treatment strategies for airway involvement in relapsing polychondritis: a comprehensive review

**DOI:** 10.3389/fimmu.2026.1843988

**Published:** 2026-06-04

**Authors:** Pengcheng Zhou, Wei Yu, Jianli Ma, Wensheng Zhang, Yan Dong, Chengshi He

**Affiliations:** 1 Department of Respiratory Medicine, Hospital of Chengdu University of Traditional Chinese Medicine, Chengdu, Sichuan, China; 2 Clinical Medical School, Chengdu University of Traditional Chinese Medicine, Chengdu, Sichuan, China; 3 Department of Respiratory Medicine, Xiaojin County People’s Hospital, Xiaojin, China

**Keywords:** airway involvement, airway management, bronchoscopy, diagnosis, relapsing polychondritis, tracheomalacia, treatment

## Abstract

Relapsing polychondritis (RP) is a rare and complex autoimmune disease primarily affecting cartilage tissue. In particular, RP leads to inflammatory damage of airway cartilage, resulting in severe complications such as airway narrowing and tracheomalacia, which has a significant effect on patients’ quality of life and prognosis. Although the clinical manifestations of RP are diverse, airway involvement has become a focal point of research and clinical attention because of its high incidence and potential lethality. Currently, the early recognition and accurate diagnosis of airway lesions remain challenging, with imaging and bronchoscopic examinations playing a key role in the assessment of structural and functional abnormalities of the airway. Although mainstream treatment consists of traditional immunosuppressants and glucocorticoids, their efficacy is limited and side effects are significant. In recent years, interventional surgery and comprehensive airway management strategies have gradually become important adjunctive treatment methods. This paper provides a systematic review of the clinical characteristics, diagnostic techniques, and diversified treatment approaches for airway involvement in RP and evaluates the latest research progress, aiming to enhance clinical physicians’ understanding and treatment levels of airway lesions in this disease and to promote the development of individualized treatment plans, thereby improving patients’ clinical prognosis and quality of life.

## 1 Introduction

Relapsing polychondritis (RP) is a rare autoimmune disease characterized by recurrent inflammatory damage to cartilage tissues, involving various structures such as the ear cartilage, nasal cartilage, joint cartilage, and airway cartilage. Because RP has diverse clinical manifestations and lack of specific laboratory indicators, its diagnosis often faces challenges, particularly in cases involving airway involvement, where delays and misdiagnoses are particularly prominent. Airway involvement occurs in approximately 50% of patients with RP, becoming one of the important causes of mortality, and the severity of airway lesions directly affects the prognosis and survival rates of patients (1, 2).

Airway involvement presents a variety of manifestations, including airway stenosis, tracheomalacia, airway collapse, and mucosal inflammation. Patients with RP with airway involvement often exhibit symptoms such as dyspnea, cough, wheezing, and cyanosis. Some patients initially present with symptoms similar to asthma or chronic obstructive pulmonary disease, which can lead to misdiagnosis as related diseases, resulting in treatment delays and worsening of the condition (3, 4). For example, in a case series study, patients with RP were misdiagnosed with asthma due to airway symptoms, ultimately developing severe airway stenosis that required tracheostomy and bronchoscopic intervention (3). In addition, imaging findings of airway lesions include thickening of the trachea and bronchial walls, destruction of tracheal cartilage, and airway stenosis, with chest computed tomography (CT) and bronchoscopy showing high diagnostic value for airway lesions, achieving positive rates of approximately 88.9% and 85.7%, respectively (1). Emerging imaging technologies such as ^18^F-fluorodeoxyglucose (FDG) positron emission tomography (PET)/CT and ^18^F fibroblast activation protein inhibitor (FAPI) PET/CT demonstrate advantages in assessing the activity and extent of airway inflammation, providing important evidence for clinical diagnosis and disease monitoring (5, 6).

The challenges in diagnosing RP patients with airway involvement primarily stem from the nonspecific nature of clinical manifestations, lack of specific laboratory indicators, and rarity of the disease itself. Early airway symptoms may be mild and easily overlooked, and some patients experience rapid progression of airway lesions, often leading to severe airway stenosis or even respiratory failure before a definitive diagnosis is made. Endoscopic examinations such as bronchoscopy and laryngoscopy can directly visualize airway mucosal and cartilage damage, aiding in the early identification of airway involvement, but they carry high operational risks, especially in patients with significantly impaired lung function (3, 7). Thus, to improve diagnostic rates and optimize treatment plans, establishing a multidisciplinary collaborative diagnosis and treatment model by integrating clinical manifestations, imaging examinations, and endoscopic evaluations is crucial (3).

With regard to treatment, managing airway involvement in RP is complex, and there is currently no unified standard. Traditional treatment primarily involves glucocorticoids combined with immunosuppressants such as methotrexate, cyclosporine, and cyclophosphamide to control inflammatory responses and slow the progression of disease (8, 9). However, some patients show poor responses to conventional treatments, with continued deterioration of their condition leading to severe airway stenosis and tracheomalacia. Bronchial intervention treatments such as airway dilation and stent placement play an important role in alleviating airway stenosis and improving respiratory function, but stent-related complications such as granulation tissue hyperplasia and secretion retention are common, and the survival rate of patients with airway stents is lower than that of those without stents (10, 11). Recent studies have shown that biologics such as anti–tumor necrosis factor α (TNF-α) drugs, rituximab, and tocilizumab exhibit good efficacy in some patients with refractory RP; in particular, the early use of biologics may reduce severe airway damage and the need for stent placement (9, 10, 12). Treatment of pediatric patients with RP with airway involvement is even more challenging; tracheostomy is an effective means of alleviating severe dyspnea, and combining techniques such as airway balloon dilation, T-tube placement, and absorbable glucocorticoid stents can reduce surgical trauma and improve the children’s survival rate and quality of life (13, 14).

Furthermore, for critically ill patients, airway intervention treatments supported by extracorporeal membrane oxygenation (ECMO) provide a new therapy option for rescuing severe airway collapse, with some cases demonstrating that this method is safe and effective, serving as a complementary approach to traditional surgical and interventional treatments (15–17). The latest surgical attempts, such as transpleural patch tracheobronchoplasty, also offer feasible surgical strategies for patients with refractory airway stenosis (18).

In summary, RP is a rare and complex autoimmune disease, with airway involvement being one of its major causes of mortality. Airway involvement presents in various forms, and diagnosis relies on a comprehensive assessment of clinical manifestations, imaging, and endoscopy. Furthermore, it can easily be misdiagnosed as other chronic airway diseases, leading to treatment delays. Current treatment relies on glucocorticoids and immunosuppressants, and the use of biologics brings new hope for refractory patients. For patients with severe airway stenosis, airway interventions and surgical procedures provide effective rescue options. Future efforts should focus on strengthening multidisciplinary collaboration, integrating advanced imaging and interventional techniques, and developing individualized treatment plans to improve the diagnostic rates and treatment outcomes of patients with RP with airway involvement, ultimately improving prognosis (1, 3, 9, 13).

## 2 Literature search strategy

**Figure 1:**
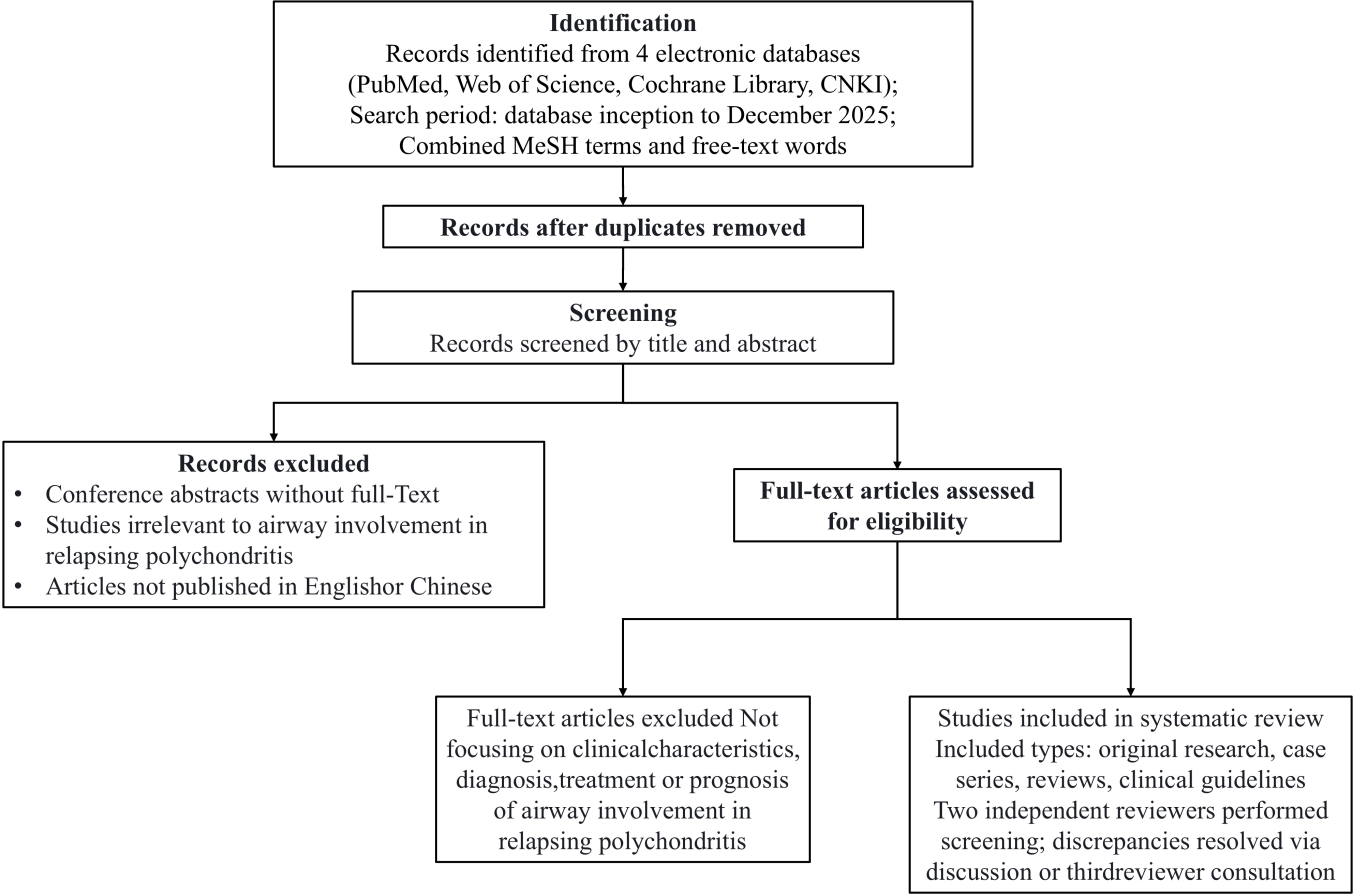
PRISMA flow diagram illustrating the literature search and selection process.

We conducted this review in accordance with the principles of systematic literature retrieval (**Figure 1**). We performed a comprehensive literature search across the following electronic databases: PubMed, Web of Science, Cochrane Library, and China National Knowledge Infrastructure. The search period covered all records from database inception to December 2025. We used a combination of Medical Subject Headings terms and free-text words to ensure comprehensive coverage.

The following search terms were used: “RP,” “airway involvement,” “tracheobronchomalacia,” “subglottic stenosis,” “bronchoscopy,” “tracheal stent,” “biological agents,” “disease-modifying antirheumatic drugs,” and “multidisciplinary team”.

Inclusion criteria were defined as follows: (1) original research, case series, reviews, or clinical guidelines focusing on the clinical characteristics, diagnosis, treatment, or prognosis of airway involvement in RP and (2) articles published in English or Chinese. Exclusion criteria were (1) duplicate publications, (2) conference abstracts without full-text availability, (3) studies not directly related to airway involvement in RP, and (4) articles not published in English or Chinese. Two reviewers independently conducted the literature-screening process, with discrepancies resolved through discussion or consultation with a third reviewer when consensus could not be reached.

## 3 Epidemiology of RP

Although RP is widely recognized clinically, its rarity poses significant challenges for large-scale epidemiological studies. The annual incidence of RP is approximately 3.5–4.5 per million, with variations in age, gender, race, and geographical distribution (19). Although RP can occur at any age, the peak incidence is primarily in middle-aged and elderly individuals between 40 and 60 years of age, with an average age of onset of about 45 to 55 years. A single-center study from the United Kingdom reported an average age of onset of 44 years (range 17–74 years) (20). Childhood onset is extremely rare, and most cases of pediatric-onset RP have been published as a single case report or a handful of case series, although the prevalence of RP is unknown (21). A family history of the autoimmune disease has been more frequently reported among children (22). With regard to gender distribution, most studies indicate a slightly higher number of female than male patients, with a male-to-female ratio of approximately 1:1.2 to 1.5. Caucasians have the highest incidence of RP, followed by individuals of African and Asian descent. One study reported that among 68 patients with RP, 81% were Caucasian, 12% were Afro-Caribbean, and 6% were Asian (20). Geographically, the highest number of cases have been reported in Northern Europe and North America, which may be related to genetic susceptibility (such as HLA-DR4 allele frequency) and environmental factors (such as smoking and infections). In contrast, lower incidence rates have been in Southern Europe and Asia, but the proportion of airway involvement is higher. A study from China reported that among 295 patients with RP, the incidence of airway involvement was as high as 70.5%, which was significantly higher than the approximately 50% reported in Europe (23). A Japanese study also reported a similarly high proportion, with an airway involvement incidence of 68% (24).

Clinical manifestations of airway involvement also vary among different races; for example, Asian patients are more prone to diffuse airway stenosis involving the main bronchi, whereas Caucasians more commonly exhibit focal laryngeal involvement (23). In recent years, with the development of international multicenter registry studies and updates to diagnostic criteria, a deeper understanding of the epidemiological characteristics of RP and its airway involvement has been achieved. However, there remain many controversies regarding the differences in incidence among different races, regions, and genders; independent risk factors for airway involvement; and long-term prognosis data. To fully reveal the disease landscape, future research must go beyond simple incidence reports and establish a globally unified epidemiological surveillance system incorporating racial stratification and detailed phenotypic analysis.

## 4 Clinical characteristics of airway involvement in RP

### 4.1 Clinical manifestations and pathological mechanisms

Airway involvement is a common and critical manifestation in patients with RP, primarily presenting clinically as dyspnea, cough, wheezing, hoarseness, and shortness of breath (**Table 1**). Cyanosis and respiratory failure may occur in severe cases, potentially threatening life. Multiple studies have reviewed the clinical features of airway involvement in patients with RP, finding that airway symptoms tend to progressively worsen and are often initially misdiagnosed as asthma or chronic cough, resulting in delayed diagnosis. For example, a PET/CT study covering 52 patients with RP with airway involvement showed that 94.2% of patients had FDG-avid lesions in the airway, suggesting persistent inflammatory activity in the airway cartilage, which was positively correlated with respiratory function indicators (such as FEV1%pred) and serum inflammatory markers (C-reactive protein (CRP), erythrocyte sedimentation rate (ESR)), further confirming the presence and severity of airway inflammation (5). In addition, pediatric patients with RP with airway involvement also display stridor, dyspnea, and hoarseness, with some experiencing significant nighttime respiratory distress, and a few may require tracheostomy to maintain airway patency (25).

**Table 1:** Clinical and imaging features of airway involvement in relapsing polychondritis.

Category	Specific item	Characteristic manifestations	Diagnostic value
I. Clinical manifestations	1. Core respiratory symptoms	Progressive Dyspnea: Worsens with exertion, may be induced or aggravated in supine position (related to airway malacia). Chronic Cough: Often persistent and dry. Wheezing and Stridor: Inspiratory or biphasic. Hoarseness: Indicates laryngeal or subglottic involvement.	Indicates severity but is easily misdiagnosed (often as asthma/COPD).
	2. Characteristic ENT signs	Auricular Chondritis: Redness, swelling, tenderness in acute phase; may cause deformity later. Laryngeal Involvement: Restricted vocal cord movement, subglottic stenosis. Saddle Nose Deformity.	Auricular chondritis is highly specific, a key diagnostic clue. However, it may be absent in those with isolated airway involvement.
	3. Systemic Manifestations	Systemic Inflammatory Symptoms: Fever, fatigue, weight loss. Multisystem Inflammation: Arthritis, scleritis/episcleritis, hearing loss, cutaneous vasculitis, cardiovascular inflammation (aortitis, etc.).	Indicates systemic disease, guiding treatment intensity. RP associated with VEXAS syndrome often has hematological abnormalities.
II. Imaging and Functional Assessment	1. Chest High-Resolution CT (HRCT)	Direct Signs: Diffuse, concentric thickening of tracheal/bronchial walls (>2mm), mainly anterior/lateral (cartilaginous); calcification/destruction of cartilaginous rings; long-segment smooth stenosis. Dynamic Assessment: Expiratory luminal collapse >50% diagnoses tracheobronchomalacia (TBM).	First-choice structural assessment tool, high sensitivity. 3D reconstruction aids surgical planning.
	2. Functional Metabolic Imaging	18F-FDG PET/CT: Shows “tramline” diffuse, symmetric FDG uptake along airways. 18F-FAPI PET/CT: Potentially more sensitive for cartilage damage/fibrosis.	Excellent for assessing inflammatory activity. Used for early diagnosis (before structural changes), assessing extent, and monitoring treatment response.
	3. Bronchoscopy	Findings: Diffuse mucosal erythema and edema; loss of cartilaginous ring markings; expiratory airway collapse (direct evidence of TBM); multiple segmental stenoses.	Gold standard for diagnosis and a therapeutic platform. Allows biopsy and intervention. High risk in severe stenosis; low FVC% is a risk factor.
	4. Pulmonary Function Tests	Flow-Volume Loop (FVL): Shows inspiratory and/or expiratory plateau, indicating fixed or variable large airway obstruction.	Important non-invasive screening tool. Typical changes may precede symptoms and CT abnormalities. Useful for assessing obstruction severity and follow-up.
III. Key Differential Diagnoses	Main Diseases for Differentiation	Granulomatosis with Polyangiitis (GPA): With pulmonary nodules, renal disease, positive c-ANCA, irregular stenosis. Tracheobronchial Amyloidosis: CT may show calcification/ossification; biopsy Congo red positive. Post-infectious/Post-intubation Stenosis: Localized lesion, clear history. VEXAS Syndrome: More common in elderly males, with hematological abnormalities, stronger inflammation.	RP is a diagnosis of exclusion. Requires comprehensive differentiation based on clinical features, serology, imaging (RP shows “diffuse smooth thickening”), and pathology.

From a pathological mechanism perspective, airway involvement in RP is primarily due to repeated inflammation of the cartilage, leading to destruction of the tracheal and bronchial cartilage, which subsequently causes airway narrowing and tracheomalacia. Tracheal and bronchial cartilage destruction results in a loss of stability in the airway structure, leaving the airway prone to collapse during exhalation, thereby exacerbating respiratory dysfunction. CT imaging studies have found that the airway cartilage in patients with RP exhibits erosion, thickening, and mucosal hyperplasia, with a rate of tracheal involvement that exceeds 80%. In addition, laryngeal involvement is relatively common, particularly erosion of the cricoid cartilage, which is the main cause of airway narrowing (23). As the disease progresses, destruction of the airway cartilage can lead to tracheobronchial softening, characterized by weakened airway walls and decreased elasticity, resulting in pronounced symptoms of airway collapse that further aggravate dyspnea (4, 15).

Genetic susceptibility and variations in genes related to inflammatory pathways further elucidate the pathogenesis of RP and the causes of airway involvement. Multiple studies have shown that human leukocyte antigen (HLA) DR4 (*HLA-DR4*) (especially HLA-DRB1*16:02, HLA-DQB1*05:02, and HLA-B*67:01) is a susceptibility factor for RP, triggering autoimmunity by affecting antigen presentation (26–28). Non-HLA genes such as protein tyrosine phosphatase nonreceptor type 22 (*PTPN22*) are also involved in the disease, and the frequency of HLA-DQB1*03:01 may be elevated in subgroups with airway involvement (28). MMP-9 and MMP-13 promoter polymorphisms accelerate the degradation of cartilage and weaken airway structure. Genetic mutations in proinflammatory effectors such as tumor necrosis factor-alpha (*TNF-α)*, interleukin-1 beta (*IL-1β*), and interleukin-6 (*IL-6*) exacerbate airway inflammation, whereas genetic mutations in the *NF-κB* pathway affect the intensity of inflammation and disease progression (28). In patients with RP, interferon-induced genes (*IFIT1, MX1*) and chemokines (*CXCL9, CXCL10*) are upregulated, suggesting activation of the type I interferon pathway (28). Local downregulation of the type II collagen gene (*COL2A1*) and upregulation of the type I collagen gene (*COL1A1*) in the airways indicate abnormal myocardial fibrosis. Levels of anti–type II collagen antibodies and anticartilage oligomeric matrix protein antibodies are positively correlated with airway stenosis (28).

It is noteworthy that patients with RP with airway involvement often present with fewer lesions in the auricle and eyes, suggesting clinical heterogeneity. Some studies indicate that patients with airway involvement have a lower incidence of auricular inflammation, are diagnosed at a younger age, have prolonged diagnosis times, exhibit poorer treatment responses, and have relatively unfavorable prognoses (1, 23). Moreover, airway involvement in patients with RP is often associated with recurrent respiratory infections due to structural damage to the airway and reduced mucosal defense function, which increases the risk of infection, further exacerbating airway damage (23). Therefore, clinical vigilance of airway symptoms in RP patients is essential, and timely examinations such as CT, bronchoscopy, and PET/CT should be performed to clarify the extent and severity of airway involvement. Early diagnosis and intervention are crucial for improving patient prognosis (5, 7).

### 4.2 Differences between pediatric and adult patients

RP is a rare immune-mediated disease that primarily affects the cartilage structures, especially the cartilage of the ears, nose, and airway, leading to progressive destruction. There are significant differences between pediatric and adult patients in the presentation and disease course of airway involvement, and understanding these differences is crucial for early identification and treatment. Pediatric patients typically present with acute respiratory symptoms, such as severe cough, hoarseness, and difficulty breathing. The condition progresses rapidly with severe airway narrowing, often requiring emergency tracheostomy due to airway obstruction. A 12-year-old patient with severe respiratory distress due to airway inflammation required intubation followed by tracheostomy, demonstrating the acute and critical nature of airway involvement in children and reflecting the severity and urgency of airway involvement in pediatric patients with RP (29).

In contrast, airway lesions in adult patients tend to progress chronically, with symptoms being subtle, often leading to delayed diagnosis, and can be misdiagnosed as other respiratory diseases. Due to the slow progression of lesions, adult patients with RP have atypical manifestations of airway involvement, often being discovered in only the later stages of the disease, which increases the risk of irreversible airway damage. Therefore, the diagnosis and management of adult patients rely more on detailed clinical evaluation and imaging studies. Due to the typically rapid and severe progression of airway lesions in pediatric patients, the need for airway intervention and surgical procedures is significantly higher than in adults. To prevent severe airway damage and improve prognosis, early diagnosis and timely treatment are crucial (29).

## 5 Diagnostic methods and phenotype for airway involvement in RP

### 5.1 Imaging examination

Imaging examinations play an irreplaceable and important role in the diagnosis and assessment of airway involvement in RP. Because RP primarily affects cartilage tissues, particularly airway cartilage, imaging can provide a visual display of changes in airway structures, providing key information for clinical diagnosis and treatment (**Table 1**).

**Figure 2:**
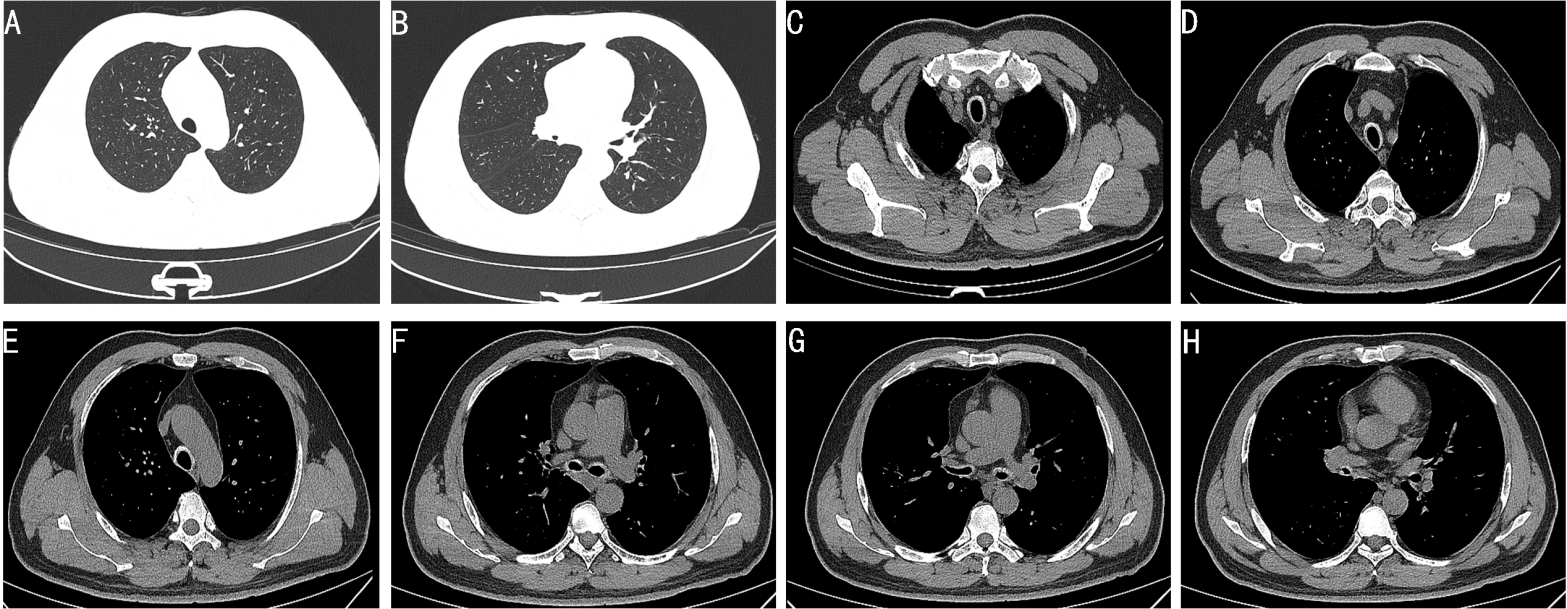
Computed tomography manifestations of airway involvement in relapsing polychondritis (RP). Images 1**(A–B)** show the lung window of patients with RP, with no significant abnormal findings. Images 1**(C–H)** show the mediastinal window, in which diffuse circumferential thickening of the trachea, left and right main bronchi, and right middle segment bronchus is observed, accompanied by localized calcification of the airway cartilage.

CT and three-dimensional reconstruction techniques are fundamental methods for assessing airway lesions in RP. CT can clearly show characteristic changes such as thickening of the airway walls, calcification, cartilage destruction, and airway narrowing (24) (**Figure 2**). Studies have shown that the tracheal and bronchial walls in patients with RP typically exhibit irregular thickening, accompanied by the destruction of the cartilage ring structure, leading to narrowing of the airway lumen and, in severe cases, even airway obstruction (30). Three-dimensional reconstruction technology can provide a three-dimensional view of the airway, which helps to more accurately assess the extent and degree of airway narrowing, which is of significant value for preoperative planning and evaluating treatment outcomes. In addition, chest CT can be used to differentiate RP from other airway diseases, such as granulomatosis with polyangiitis (GPA), which often affects the subglottic trachea and extensive airways, whereas RP is more characterized by the thickening of the tracheal cartilage rings, with less involvement of the soft tissue of the posterior wall (31).

Dynamic expiratory CT plays an important role in the evaluation of tracheomalacia and airway collapse. Tracheomalacia occurs in patients with RP due to cartilage destruction, making the airway prone to collapse during expiration and resulting in breathing difficulties and wheezing. Dynamic expiratory CT can capture the dynamic changes in the airway during expiration, showing the degree and extent of collapse of the trachea and bronchi and providing a basis for the quantitative assessment of airway dysfunction (3). This technology can identify early manifestations of tracheomalacia, providing guidance for clinical decisions regarding the appropriate airway support or interventional treatment.

^18^F-FDG PET/CT, as a metabolic imaging technique, can reflect the activity of inflammation. In RP, active inflammatory cell infiltration is usually present at the sites of airway lesions, leading to increased local glucose metabolism, which is manifested as enhanced FDG uptake. Several studies have reported that ^18^F-FDG PET/CT can show areas of active inflammation in the trachea, bronchi, and other cartilage sites, aiding in the early diagnosis of RP and differentiating it from noninflammatory airway diseases such as asthma (5, 32, 33). Furthermore, PET/CT is also valuable in the assessment of treatment responses, as a decrease in the FDG uptake in areas of active inflammation suggests remission. Notably, recent studies suggest that ^18^F-FAPI PET/CT may outperform ^18^F-FDG PET/CT in the diagnosis of RP, as it is more sensitive in showing cartilage damage and fibrosis, providing more precise information for clinical management (6).

In summary, the combined application of various imaging techniques can comprehensively and accurately assess airway involvement in RP, guiding the formulation of individualized clinical treatment strategies, reducing the incidence of airway complications, and improving patient prognosis.

### 5.2 Bronchoscopy

**Figure 3:**
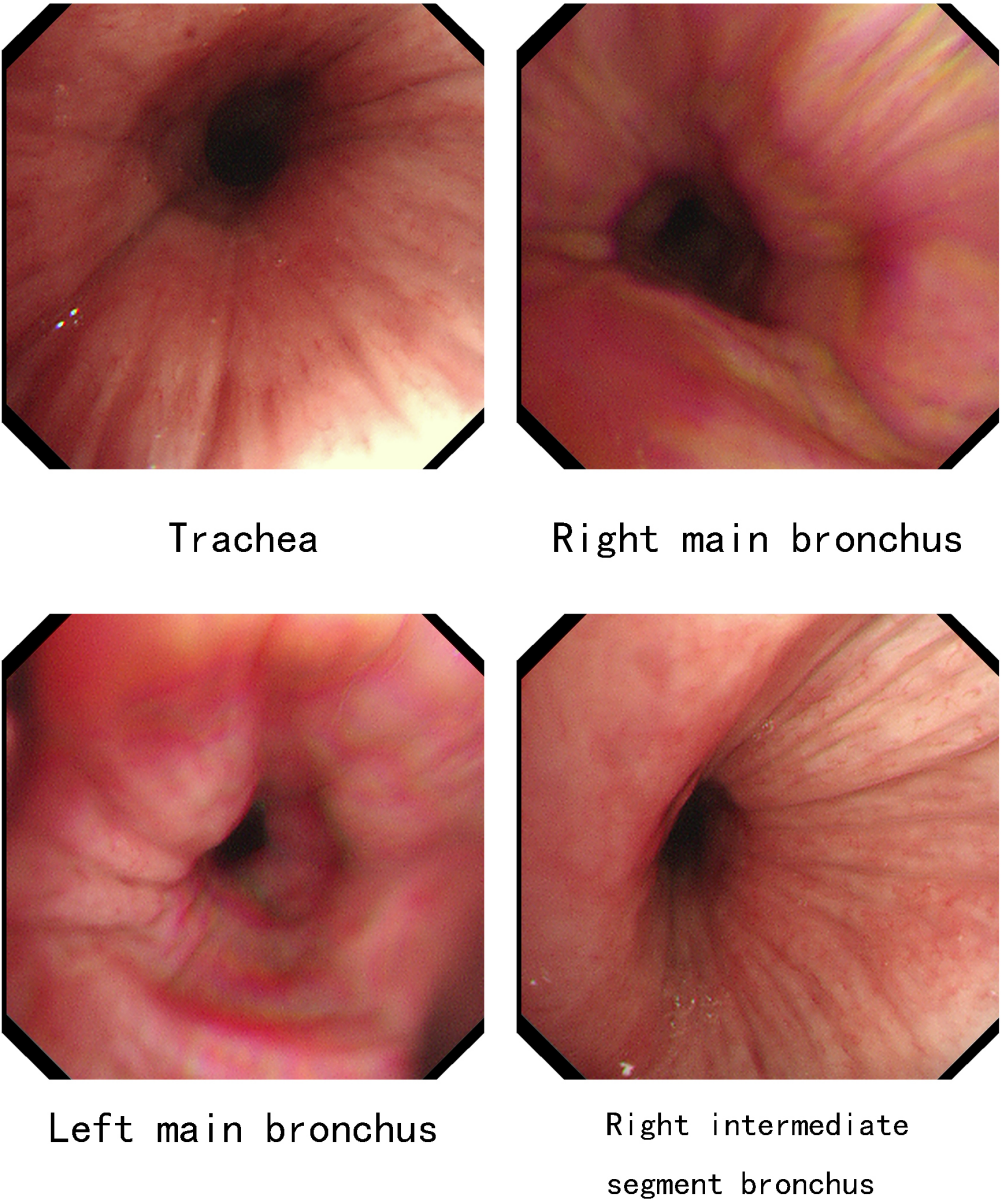
Bronchoscopic findings of airway involvement in relapsing polychondritis. The images show congestion and swelling of the mucosa in the trachea, left and right main bronchi, and right middle segment bronchus, with disappearance of the cartilage rings and narrowing of the lumen.

Bronchoscopy plays an irreplaceable role in the diagnosis of airway involvement in RP and is regarded as the gold standard for diagnosing airway involvement. The bronchoscope allows for direct observation of inflammatory manifestations of the airway mucosa, exposure of cartilage, and the specific degree of airway narrowing, thus providing an intuitive basis for the clinical assessment of disease activity and extent of involvement. In patients with RP, airway involvement often manifests as inflammation and destruction of the airway cartilage, leading to softening of the airway cartilage (tracheobronchomalacia) and severe airway narrowing (**Figure 3**). During bronchoscopy, the airway mucosa may show congestion, edema, and even characteristic changes such as mucosal roughness and nodular alterations (1, 7). Furthermore, bronchoscopy can also identify dynamic changes in airway collapse, absence of cartilage rings, and mucosal lesions, providing dynamic imaging evidence for disease assessment (4, 34).

Bronchoscopy not only allows for the direct visualization of lesions but also enables biopsy during the procedure, facilitating the acquisition of pathological tissue from the airway mucosa and cartilage, which is crucial for a definitive diagnosis. Biopsy specimens can reveal pathological features such as infiltration of inflammatory cells, cartilage destruction, and fibrous tissue hyperplasia, which can be used to rule out other inflammatory or neoplastic lesions (8, 35). Especially when airway symptoms are atypical and imaging studies are inconclusive, bronchoscopy-assisted biopsy can improve the diagnostic accuracy and early diagnosis rates, avoiding misdiagnosis as asthma or chronic obstructive pulmonary disease (3, 36).

It is worth noting that bronchoscopy carries certain risks in patients with RP, especially those with severely impaired lung function. Studies have shown that low forced vital capacity (FVC%) is an independent risk factor for serious adverse events during bronchoscopy; therefore, thorough assessment of the patient’s lung function is necessary prior to the procedure, along with preparation for emergency intubation or tracheostomy measures (34). In addition, bronchoscopy should be performed by experienced physicians to avoid airway obstruction or other complications due to improper handling (37).

### 5.3 Pulmonary function

RP with airway involvement exhibits a series of characteristic changes that directly reflect the destruction of the airway cartilage structure and functional abnormalities. Restrictive ventilatory dysfunction is a common manifestation of RP airway involvement, with the core mechanism being airway stenosis and dynamic collapse leading to gas trapping, which in turn causes a reduction in lung volume, which manifests as decreased FVC and total lung capacity (38). Meanwhile, airway resistance is significantly increased, which can be detected using impulse oscillometry (IOS). Studies have shown that total respiratory impedance (Zrs) and parameters reflecting central and peripheral airway resistance (such as R5 and R20) are elevated in patients with RP, especially during the expiratory phase, which is a direct indication of the presence of dynamic airway collapse (24). Morphological changes in the maximal expiratory flow-volume curve are of important suggestive value, often presenting as a “bimodal” or “plateau” pattern. This pattern indicates variable airway obstruction, in which both inspiratory and expiratory flows are limited but expiratory limitation is more pronounced (39). It is worth noting that unless combined with pulmonary parenchymal lesions or secondary pulmonary hypertension, the diffusing capacity of the lung for carbon monoxide inpatients with RP usually remains normal or only mildly decreased (40). Although a single lung function result cannot definitively indicate airway involvement in RP patients, the comprehensive use of conventional lung function tests and IOS can provide important evidence for the early identification and severity assessment of airway involvement in RP.

### 5.4 Laboratory examination

Although laboratory tests do not provide specific indicators, they hold significant value in aiding diagnosis and assessing the disease. Levels of inflammatory markers such as ESR and CRP are commonly elevated and correlate with disease activity, with the CRP to albumin ratio identified as an independent risk factor used for assessing RP activity (41). In immunologic testing, some patients may have detectable autoantibodies such as anti–type II collagen antibodies, but the sensitivity and specificity of this are limited, and a routine diagnostic standard have not been established (42). In addition, hematologic abnormalities such as megaloblastic anemia, thrombocytopenia, and lymphopenia are particularly prominent in patients with RP associated with VEXAS (vacuoles, E1 enzyme, X-linked, autoinflammatory, somatic) syndrome, indicating the need for differential diagnosis based on clinical features (43, 44).

### 5.5 Airway involvement phenotype

Based on the location and nature of the lesions, airway involvement in RP can be classified into four main phenotypes. The most common is the subglottic stenosis phenotype, accounting for approximately 50% to 60% of patients with airway involvement (45). Chest CT scans can reveal circumferential or eccentric stenosis in the subglottic region (at the level of the cricoid cartilage), manifesting as airway wall thickening, calcification, or cartilage destruction (46). Bronchoscopy may show mucosal congestion, edema, or scar formation (37). Another important phenotype is the main bronchomalacia phenotype, characterized by softening of the cartilage in the trachea and main bronchi, leading to dynamic collapse. A significant reduction in airway diameter during expiration can be observed on expiratory phase CT or dynamic bronchoscopy, with patients often presenting with expiratory dyspnea (7). In addition, the diffuse tracheobronchial stenosis phenotype involves extensive fixed stenosis from the trachea to the segmental bronchi, with CT showing diffuse thickening of the airway wall and irregular luminal narrowing (47). These phenotypes often coexist clinically, such as subglottic stenosis combined with main bronchomalacia, forming a mixed phenotype that poses greater treatment challenges.

## 6 Diagnosis and differential diagnosis

**Figure 4:**
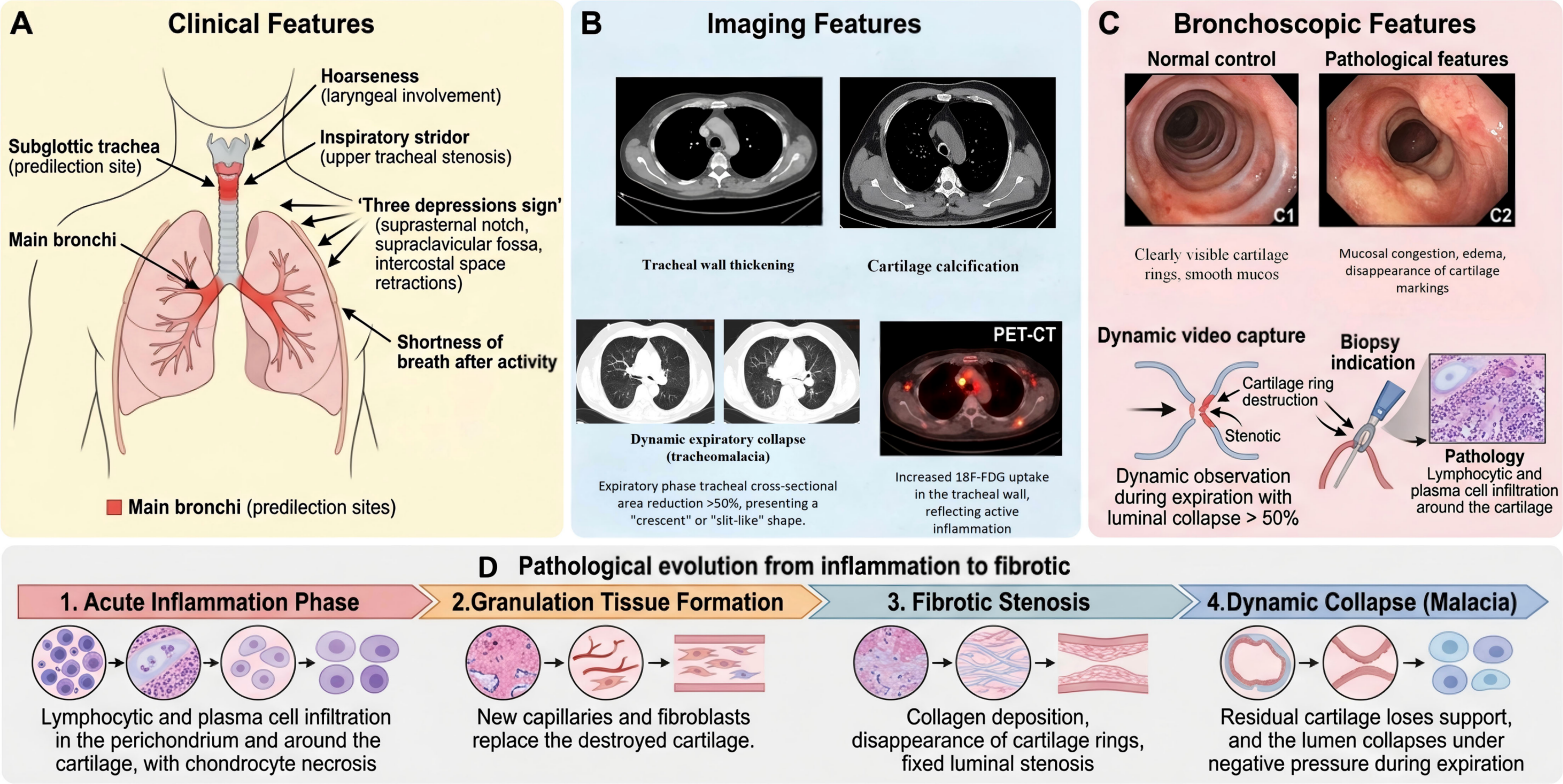
Key clinical, radiologic, endoscopic, and pathological features of airway involvement in relapsing polychondritis. **(A)** Clinical manifestations. **(B)** Imaging findings. **(C)** Bronchoscopic features. **(D)** Pathological progression from acute inflammation to fibrotic stenosis and dynamic collapse.

The diagnostic criteria for RP have evolved from classic to integrated approaches, with the aim of improving diagnostic sensitivity and specificity. The classic McAdam diagnostic criteria (1976) require at least three clinical features, such as bilateral auricular chondritis, nonerosive seronegative polyarthritis, nasal chondritis, ocular inflammation, respiratory tract chondritis, and cochlear or vestibular dysfunction (48). These criteria have high specificity but lack sensitivity for early or atypical cases, especially those with airway involvement as the initial or predominant manifestation, leading to potential missed diagnoses (49). Subsequent revised diagnostic criteria (e.g., Damiani and Levine criteria) have added histopathological evidence (cartilage biopsy showing chondritis) and response to glucocorticoid therapy as auxiliary conditions, enhancing diagnostic flexibility (49). The new criteria proposed by the International RP Study Group in 2015 place greater emphasis on integrating imaging evidence (e.g., CT showing tracheal wall thickening, calcification, or collapse) and serological markers (e.g., anti–type II collagen antibodies), which is particularly applicable to cases with predominant airway involvement (48). A high index of suspicion is required for airway involvement; even if systemic symptoms are atypical, comprehensive assessment should combine bronchoscopy (revealing airway mucosal congestion, loss of cartilage rings, and dynamic luminal collapse) with the aforementioned imaging examination abnormalities (50) (**Figure 4**). Early diagnosis and intervention are crucial for improving the prognosis of RP, especially in patients with airway involvement.

The involvement of the respiratory tract in RP requires differentiation from various diseases that cause large airway stenosis, with the key being to grasp their characteristic manifestations. First, it must be distinguished from GPA. GPA is often accompanied by ulcers of the upper respiratory tract, kidney involvement, and positive c-ANCA, whereas ANCA is typically negative in RP. Radiologically, airway lesions in GPA are mostly focal ulcers or nodules, whereas RP presents with diffuse tracheal wall thickening and calcification (48). Second, RP should be differentiated from tracheobronchial amyloidosis. The latter disease is characterized by diffuse or nodular amyloid deposits in the airway wall, with calcifications visible on CT, but it lacks the characteristic cartilage destruction and dynamic collapse of RP as well as systemic manifestations such as auricular and nasal chondritis (51). Immunoglobulin G4 (IgG4)–related disease is a fibroinflammatory condition characterized by elevated serum IgG4 levels, extensive infiltration of IgG4-positive plasma cells in tissues, and pathological features such as storiform fibrosis and obliterative phlebitis (52). It can affect any part of the body, including airway involvement (53). Although imaging and endoscopic findings do not show obvious specificity, positive IgG4-specific staining is an important basis for diagnosis (54). Tracheobronchopathia osteochondroplastica (TO) is characterized by multiple submucosal bony nodules in the airway, with CT showing a “beaded” appearance, but there is no evidence of inflammatory activity, and pulmonary function typically does not exhibit dynamic collapse (55).

VEXAS syndrome and RP exhibit significant overlap and differences in chondritis-related manifestations. Both can involve the auricle, nasal, and respiratory tract cartilage, presenting as auricular redness, swelling, pain, and deformity; saddle nose deformity; and tracheobronchial chondritis, leading to stenosis and dyspnea (56),. VEXAS is a clonal hematopoietic disorder driven by a clear somatic mutation in the UBA1 gene, constituting a monogenic disease (57). In the vast majority of VEXAS patients, mutations occur at the p. Met41 site of this gene, resulting in aberrant RNA splicing and loss of enzymatic activity (58). The immunopathological mechanism of RP is more complex and has traditionally been considered an autoimmune response that primarily targets cartilage components. Autoantibodies against type II, IX, and XI collagen, as well as cartilage oligomeric matrix protein, can be detected in patients (59). However, chondritis in VEXAS syndrome is typically more persistent and difficult to control, showing poor response to conventional immunosuppressive therapy (57). VEXAS syndrome should be highly suspected in patients presenting with high-risk features, such as elderly men (usually those older than 50 years), accompanied by macrocytic anemia, thrombocytopenia, characteristic skin rashes (e.g., Sweet syndrome–like lesions), fever, pulmonary infiltrates, or refractory disease, and UBA1 gene testing should be prioritized (43). In the standard evaluation process for all patients with suspected RP, UBA1 gene testing should become an important future pathway.

In addition, RP must be differentiated from infectious tracheitis (e.g., tuberculosis, fungal infections), sarcoidosis, and malignant tumors (e.g., tracheal adenoid cystic carcinoma) (48). Etiological examination, bronchoscopic biopsy, and imaging methods such as PET-CT can be used to distinguish RP from malignant tumors (2). Comprehensive differential diagnosis relies on detailed medical history, physical examination, and multimodal auxiliary examinations to avoid misdiagnosis and delayed treatment.

## 7 Treatment for airway involvement in RP

The treatment of RP airway lesions presents a clear “stepwise integration” framework. Pharmacotherapy, particularly corticosteroids combined with immunosuppressants, forms the cornerstone of systemic anti-inflammatory therapy for all phenotypes. However, the diffuse stenosis phenotype often exhibits stronger inflammatory activity and resistance to conventional treatments, highlighting the need for the early introduction of biologics. Noninvasive ventilation therapy can function as a pneumatic stent and may serve as a foundational treatment. At the level of interventional and surgical therapy, decision-making is highly dependent on the anatomical characteristics of the stenosis. For focal, circumferential stenoses (e.g., partial subglottic stenosis), balloon dilatation and stent placement can effectively restore luminal patency, whereas for long-segment, complex malacia or mixed lesions, tracheal sleeve resection, tracheoplasty, and even tracheostomy may be crucial measures for maintaining airway safety. The key point is that any local intervention must be conducted concurrently with adequate systemic immunosuppressive therapy; otherwise, the restenosis rate is extremely high, which profoundly reflects the complexity of RP as a systemic disease.

### 7.1 Glucocorticoids and immunosuppressants

Glucocorticoids are the first-line treatment for airway involvement in RP, capable of rapidly controlling inflammatory responses and alleviating clinical symptoms such as dyspnea. Relevant studies indicate that glucocorticoids play a key role in RP treatment, with their quick anti-inflammatory effects contributing to the control of the inflammatory destruction of airway cartilage and delay in disease progression (8). In clinical practice, high-dose glucocorticoid treatment is often used during acute exacerbations, followed by gradual tapering to maintain efficacy and reduce the occurrence of glucocorticoid-related side effects (60). However, when treated with glucocorticoids alone, some patients may face issues of disease relapse and glucocorticoid dependence, and long-term high-dose use can also lead to adverse reactions such as osteoporosis and hyperglycemia.

Therefore, a treatment strategy that combines immunosuppressants has been widely adopted to enhance efficacy and reduce the use of glucocorticoids. Commonly used immunosuppressants include methotrexate, cyclophosphamide, azathioprine, and mycophenolate mofetil (1, 10). Methotrexate, as a first-line immunosuppressant, effectively controls cartilage inflammation, reduces the progression of airway narrowing, and significantly decreases the relapse rate (25). Cyclophosphamide is mainly used for cases with severe airway involvement or multiorgan involvement, and its potent immunosuppressive effect helps control disease activity; however, due to its potential toxic side effects, it requires strict monitoring (1). The combined use of glucocorticoids and immunosuppressants not only enhances the anti-inflammatory effect but also reduces the amount of glucocorticoids used, lowering the risk of glucocorticoid-related side effects and improving patients’ quality of life (61).

Despite this, some patients do not respond well to traditional immunosuppressants, showing difficulties in disease control or frequent relapses, indicating certain limitations in the current treatment model (1). In addition, recent studies on RP-related to VEXAS syndrome indicate that patients with bone marrow abnormalities have poorer responses to traditional immunosuppressive therapy and a higher risk of relapse, suggesting the need for individualized treatment plans (44, 62).

### 7.2 Application of biologics

In recent years, the application of biologics in the treatment of RP, especially in refractory patients, has garnered increasing attention. Biologics such as TNF-α inhibitors (adalimumab) and rituximab have shown good therapeutic effects, particularly after the failure of traditional immunosuppressants, making them important second-line treatment options. According to reports in the literature, TNF-α inhibitors are among the most commonly used biologics in patients with RP, with some cases demonstrating complete or partial remission. However, it is important to note that efficacy may diminish over time. In some cases, infliximab and adalimumab are recommended as preferred options (9, 63). In addition, rituximab, as an anti-CD20 monoclonal antibody, has shown certain efficacy in some refractory cases, but its effectiveness is controversial, and it is not recommended as a first-choice biologic (9, 64).

The early use of biologics in patients with airway involvement in RP may bring significant clinical benefits. Existing studies have shown that early intervention with biologics can delay the progression of airway lesions and reduce the severity of airway cartilage destruction and airway collapse, thereby decreasing the rate of airway stent implantation. Specific research data indicate that in patients treated with biologics, the rate of airway stent implantation is significantly lower than that in those not using biologics, and the survival rate of patients in the biologics group is higher (10). Furthermore, a multicenter retrospective study found that about half of patients with RP received biologic therapy, and these patients often had more severe airway damage, such as subglottic stenosis and tracheomalacia. The early and rational use of biologics helps control inflammation and reduce the incidence of airway complications (20).

Although biologics demonstrate good therapeutic potential in the treatment of RP, their safety and long-term efficacy require further research and validation. The existing literature has reported adverse events related to different biologics, such as tocilizumab potentially exacerbating intestinal symptoms in patients with concomitant ulcerative colitis, indicating that caution should be exercised in patients with concomitant bowel diseases (65). Moreover, issues such as infection risk and diminishing efficacy during biologic use still need to be addressed; some patients may develop resistance to treatment or lose responsiveness. There have also been reports indicating that IL-17 inhibitors such as secukinumab have achieved successful remission in some refractory RP patients, but such experiences are still anecdotal and lack the support from large-scale data (66). Therefore, the current clinical application of biologics relies more on case experience and individualized assessment, necessitating systematic clinical trials to determine the optimal timing, dosage, and duration of use to ensure safe and effective treatment (9, 67).

### 7.3 Noninvasive airway support (continuous positive airway pressure)

Noninvasive mechanical ventilation (NIV) is an important treatment for respiratory failure resulting from RP airway involvement, with indications including daytime hypercapnia, nocturnal hypopnea, and inspiratory stridor. In patients with laryngeal collapse, the early application of NIV can delay tracheostomy and maintain airway patency through positive-pressure support. During the acute exacerbation stage, NIV should be initiated urgently, but tolerance must be monitored; in cases of altered consciousness or hemodynamic instability, conversion to mechanical ventilation is required. When sleep apnea is present, parameter adjustment should be cautious. The bilevel positive airway pressure mode is preferred, with typical settings of IPAP 12 to 20 cmH_2_O and EPAP 4 to 8 cmH_2_O; The average volume-assured pressure support mode can be used for dynamic collapse. Parameter adjustments must be individualized, guided by pulmonary function and blood oxygen monitoring. The efficacy evaluation includes short-term indicators (Borg score, blood gas analysis, nocturnal oxygen saturation) and long-term follow-up (pulmonary function and CT every 3–6 months). Complications such as air leakage and skin pressure ulcers should be managed by replacing the mask and humidifier. Patients who are dependent on NIV should establish a home care plan and combine immunosuppressive agents to control the underlying disease. Although NIV has a pneumatic stent effect that can maintain a certain degree of airway patency and serve as a basic treatment, for those with poor efficacy, treatment that includes a combination of medication, interventional procedures, or surgery is necessary.

### 7.4 Airway stenting

**Figure 5:**
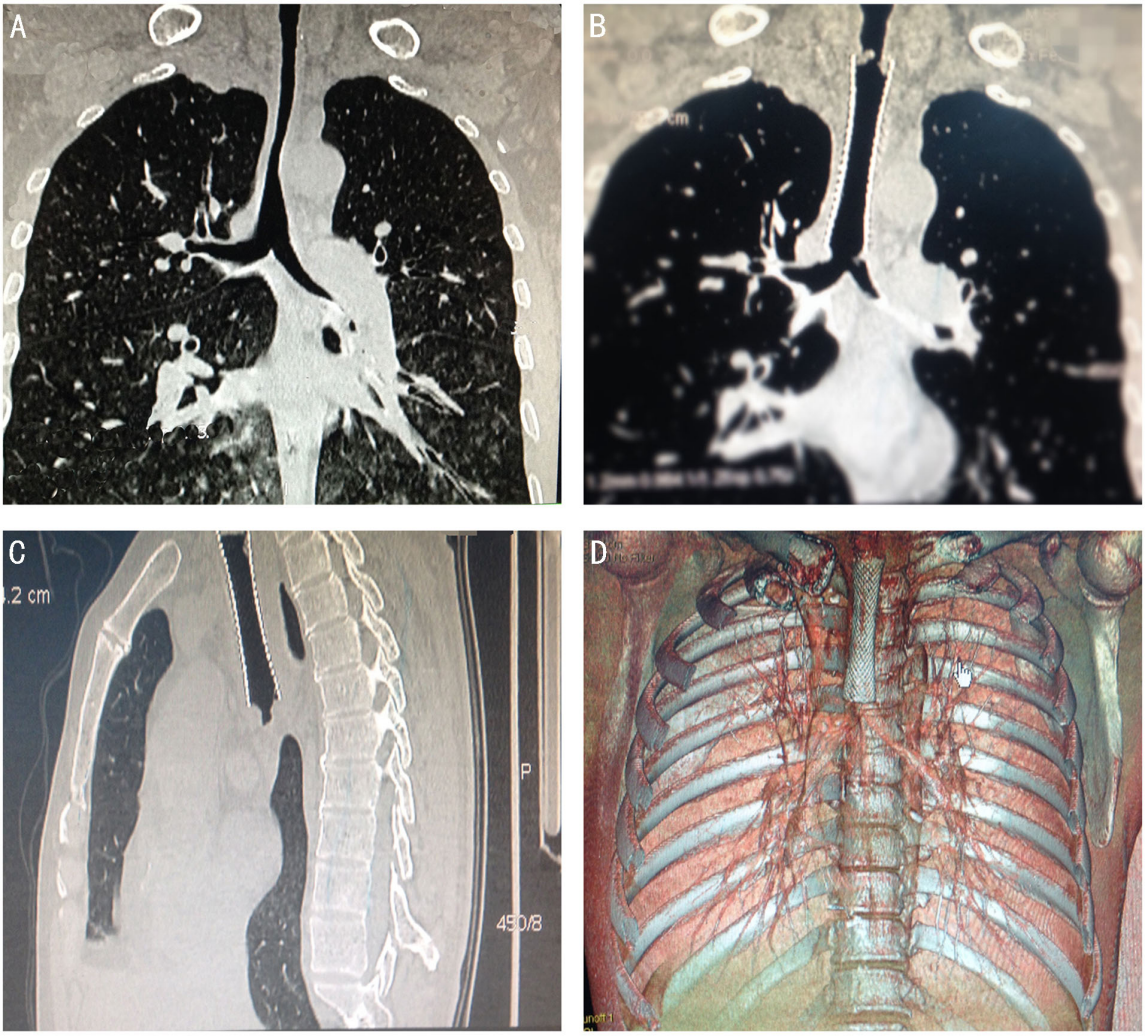
Stent implantation treatment for airway involvement in relapsing polychondritis. Image 3 **(A)** shows extensive narrowing of the trachea in the coronal view. Images 3 **(B–D)** show significant enlargement of the tracheal lumen after stent implantation.

In patients with RP, airway stenting is an important interventional method for alleviating airway stenosis and improving respiratory function (**Figure 5**). The rate of airway involvement in patients with RP can be as high as 50%, with airway stenosis and destruction of airway cartilage often resulting in respiratory difficulties and even life-threatening conditions. There are two primary types of airway stents: silicone stents and metal stents. Because of their good biocompatibility and ease of removal, silicone stents are widely used in airway reconstruction for patients with RP; metal stents are suitable for supporting more severe airway stenosis or cartilage defects because of their sturdy structure (68). In some complex cases, a combination of silicone and metal stents is used to adequately support the airway and relieve multiple stenoses, such as in a case in which a 64-year-old patient with RP underwent the sequential implantation of silicone and metal stents, significantly improving symptoms of dyspnea and achieving long-term disease stability (68).

Although airway stents are effective in improving respiratory function, multiple stent implantations are often associated with a series of complications. Common complications include granulation tissue hyperplasia, retention of mucus, stent displacement, and airway infections. Among these, granulation tissue hyperplasia is particularly common and may lead to recurrent airway stenosis, which affects the efficacy of the stents and possibly necessitates repeated stent removal or replacement (10). Mucus retention, due to the space-occupying effect of the stents and damage to the airway mucosa, can easily trigger infections and bronchial inflammatory responses, further exacerbating airway obstruction. The risk of these complications increases with multiple stent implantations, necessitating close monitoring and timely management (11).

Stent-related complications have a significant effect on patient survival rates. Clinical studies have shown that patients with RP who undergo airway stenting have a significantly higher mortality rate due to complications as compared with those who do not receive stents, which indicates the necessity of weighing the pros and cons during the selection and implantation of airway stents (10). Therefore, although airway stenting is an effective method for treating RP-related airway stenosis, decisions should be made comprehensively based on the patient’s condition, degree of airway stenosis, and systemic treatment plans, and blind multiple stent implantations should be avoided to reduce the occurrence of complications and enhance the quality of life and survival time of the patient.

### 7.5 Bronchoscopic interventional therapy

Bronchoscopic interventional therapy plays an important role in the treatment of airway involvement in RP, especially in the alleviation of airway stenosis and improvement in patient respiratory function. Common interventional techniques include balloon dilation and laser airway remodeling, which can effectively expand narrowed airways, reshape airway structures, and relieve patients’ breathing difficulties. For example, laser airway remodeling improves the patency of the airway by selectively ablating the soft tissue of the airway lesions, which reduces inflammation and mucosal edema at the stenosis site. Balloon dilation mechanically expands the narrowed segments of the airway, restoring the airway diameter and significantly improving patients’ ventilation function (69). These interventional techniques provide effective local treatment options for patients with RP and severe airway involvement, especially when the effects of medication are limited or cannot quickly relieve the airway obstruction.

For patients with severe conditions accompanied by tracheobronchomalacia, traditional methods of anesthesia and mechanical ventilation often struggle to meet the safety demands of interventional treatments. In recent years, interventional therapy combined with ECMO support has provided new safety guarantees for these patients. ECMO support allows clinicians to maintain oxygenation and ventilation in cases of extreme narrowing or even airway collapse, effectively avoiding the risk of respiratory failure during interventional procedures. Case reports have shown that patients with RP with severe airway softening and stenosis, who did not benefit from standard treatment and airway stenting, successfully underwent bronchoscopic interventional therapy with ECMO assistance for airway stent placement, significantly alleviating breathing difficulties, and these patients were discharged smoothly without significant complications, demonstrating the application prospects of this technology in complex airway lesions (15, 16).

However, interventional therapy’s effectiveness is highly dependent on early accurate diagnosis and close cooperation among multidisciplinary teams. The diagnosis of airway involvement in RP often faces challenges and requires a comprehensive assessment that uses various methods, including dynamic chest imaging, pulmonary function tests, bronchoscopic examinations, and PET/CT scans. Early diagnosis can provide a timely assessment of the degree of airway stenosis and softening, guiding the formulation of individualized treatment plans while avoiding irreversible airway damage due to delayed diagnosis (5, 70). Furthermore, collaboration among multidisciplinary teams, including rheumatology, respiratory medicine, thoracic surgery, and interventional specialists, can achieve seamless integration in medication management, interventional procedures, and postoperative care, maximizing treatment safety and efficacy while minimizing complications and recurrence rates (3).

### 7.6 Surgical treatment

Surgical treatment primarily targets patients with severe airway collapse or stenosis in the management of RP with airway involvement, especially when conservative and immunosuppressive therapies are ineffective. Tracheoplasty, tracheostomy, and tracheal reconstruction are currently the most commonly used surgical methods in clinical practice. Tracheoplasty restores airway patency by repairing and reshaping the damaged tracheal structure, tracheostomy is mainly used for patients with severe airway obstruction who urgently need an airway established, and tracheal reconstruction is suitable for cases with extensive tracheal damage or stenosis that require structural reconstruction. Clinical reports indicate that some patients with RP experience tracheal collapse due to inflammation of the airway cartilage, and after undergoing tracheostomy, they can quickly experience relief of respiratory distress symptoms and improvement in their quality of life, although postoperative airway management and prevention of complications must still be addressed (14, 71).

In recent years, new types of tracheoplasty have achieved good results in the treatment of RP-related airway lesions. For example, pericardial patch tracheobronchoplasty uses the patient’s own pericardial tissue to support the trachea, enhancing its mechanical stability and reducing the risk of postoperative recurrence. Related case reports have indicated that some patients experience significant improvement in respiratory function and effective relief of airway stenosis after undergoing such minimally invasive or modified surgeries. Meanwhile, combining auxiliary measures such as endotracheal stent placement (e.g., bioabsorbable corticosteroid stents) and balloon dilation can further reduce the likelihood of airway stenosis recurrence, improve surgical success rates, and enhance patient quality of life (13, 14).

However, because airway structures in patients with RP are often accompanied by inflammatory activity, the surgical risks are relatively high. During surgery, it is essential to rigorously assess the patient’s overall condition, degree of airway inflammation, and potential comorbidities to prevent intraoperative airway injury and postoperative infection recurrence. In addition, postoperative patients must undergo long-term follow-up to monitor airway patency and disease activity, with immunosuppressive therapy adjusted in a timely manner. Multidisciplinary team collaboration, including rheumatology, otolaryngology, and pulmonology, can effectively optimize the diagnosis and treatment process, reduce surgery-related risks, and improve patient prognosis (3, 72).

## 8 Prognosis and management strategies for airway involvement in RP

### 8.1 Prognostic factors

The prognosis of airway involvement in RP is influenced by various factors, with the main determinants being the severity of airway involvement, diagnostic delay, treatment response, and complications. First, the severity of airway involvement is a key indicator of prognosis. Studies have shown that airway softening and multiple stent placements in patients are significantly associated with lower survival rates. The more severely the airway structure is damaged, the more pronounced the decline in respiratory function, leading to an increased risk of dyspnea and respiratory failure, resulting in a naturally poorer prognosis. For example, a study on imaging and pulmonary function in patients with RP with airway involvement found that tracheobronchial volume and minimum tracheal cross-sectional area are significantly correlated with lung function, suggesting that the degree of airway structural damage directly affects respiratory function and, consequently, patient survival (24).

Second, diagnostic delay is another important factor that affects prognosis. Because of the diverse clinical manifestations of RP and the lack of specific markers, especially as airway symptoms are often misdiagnosed or overlooked, the time to diagnosis is prolonged. Relevant studies indicate that RP patients with airway involvement have a significantly longer time to diagnosis as compared with patients without airway involvement, and diagnostic delay is negatively correlated with disease progression and treatment efficacy. Delayed diagnosis often leads to the deterioration of airway lesions, increasing the risk of airway collapse and stenosis, thereby worsening the patient’s condition and reducing the effectiveness of the treatment (1).

Treatment response also significantly affects prognosis. Early diagnosis and the timely initiation of glucocorticoid and immunosuppressive therapy can effectively control inflammation, improve airway lesions, and delay the progression of disease. Clinical cases show that although airway obstruction may be difficult to completely reverse, early drug treatment can significantly improve patient symptoms and lung function, reducing respiratory complications (6, 7). In addition, for patients with severe airway stenosis, the reasonable selection of local airway management measures such as stent placement or tracheostomy can help relieve dyspnea, but the survival rate for patients who require multiple stent placements remains low, indicating a poorer prognosis for such patients (73).

The occurrence of complications further affects prognosis. Severe airway involvement is prone to airway collapse, infection, and respiratory failure, with some patients having concurrent involvement of other systems such as cardiac lesions, which increases the risk of death. Research indicates that airway-related complications are an important cause of death in patients with RP, suggesting that clinicians must closely monitor and actively manage complications (5, 74).

In summary, the prognosis for patients with airway involvement in RP depends on the severity of airway involvement, timeliness of diagnosis and treatment, treatment response, and management of complications. The early identification of airway involvement and proactive intervention, especially performing bronchoscopy and imaging assessment when symptoms are mild, combined with glucocorticoid and immunosuppressive therapy, can significantly improve patients’ survival rates and quality of life (5, 7). To further enhance prognostic levels, future efforts should strengthen the comprehensive assessment and individualized treatment strategies for patients with RP who have airway involvement.

### 8.2 Multidisciplinary comprehensive management

**Figure 6:**
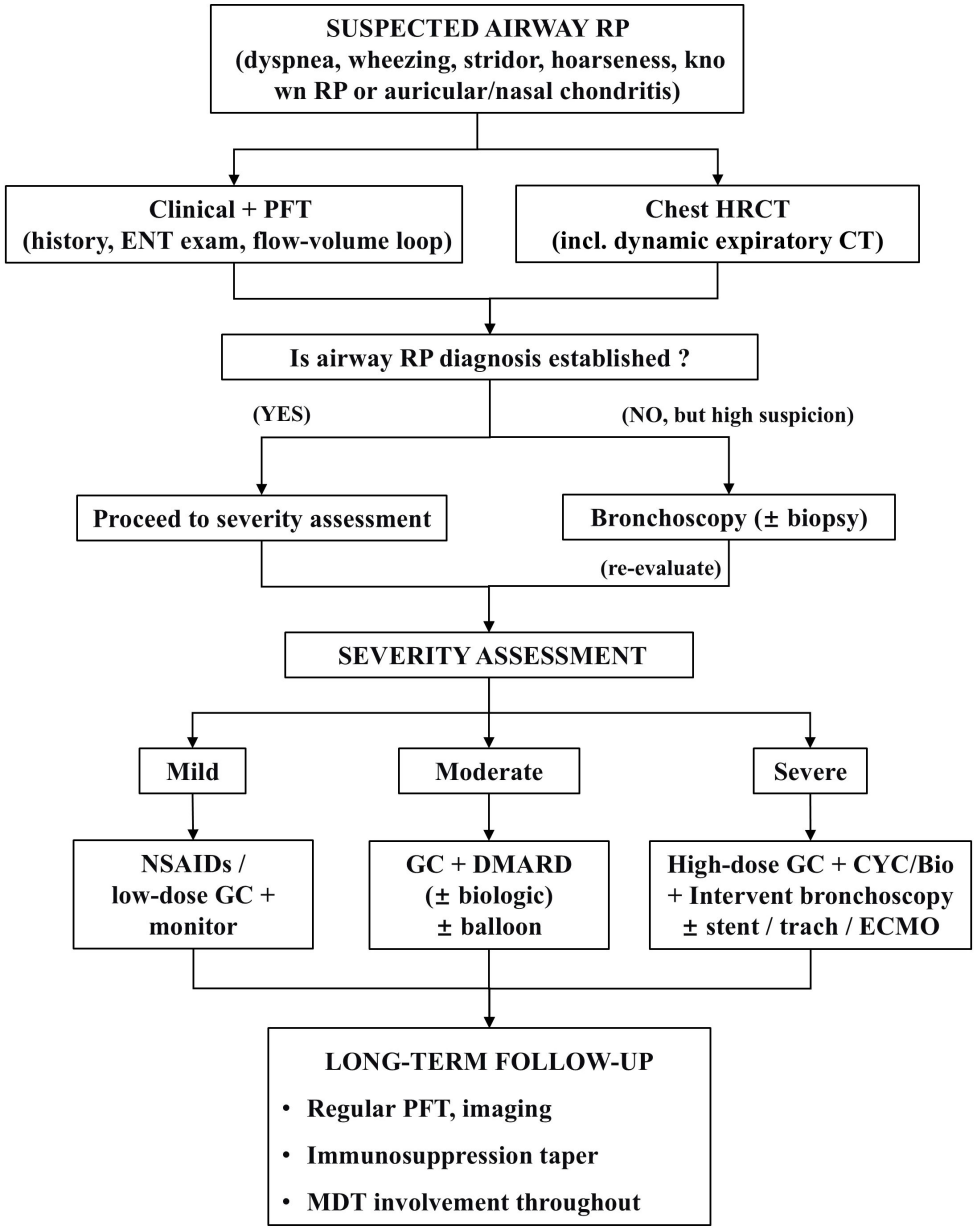
Diagnostic and therapeutic flowchart for airway involvement in relapsing polychondritis. The flowchart integrates clinical, functional, and imaging data to confirm the diagnosis, followed by severity-based treatment stratification. Mild cases receive conservative medical therapy; moderate cases add immunosuppression and consider endoscopic balloon dilation; severe cases require aggressive immunomodulation combined with interventional bronchoscopy, airway stenting, tracheostomy, or ECMO-supported procedures. Multidisciplinary team (MDT) involvement is recommended throughout the diagnostic and therapeutic pathway. RP, relapsing polychondritis; PFT, pulmonary function test; GC, glucocorticoids; MTX, methotrexate; DMARDs, disease-modifying antirheumatic drugs; CYC, cyclophosphamide; ECMO, extracorporeal membrane oxygenation; FEV1, forced expiratory volume in 1 second.

In the diagnosis and treatment of RP, multidisciplinary comprehensive management is a key strategy, especially in patients with airway involvement (**Figure 6**). RP, as a rare autoimmune systemic inflammatory disease, presents a variety of clinical manifestations and involves multiple organ systems, in particular the ear, nose, throat, respiratory system, and cartilage structures (**Table 2**). Airway involvement often results in severe respiratory dysfunction and even life-threatening situations. Thus, the collaborative efforts of rheumatology, respiratory medicine, otolaryngology, and intensive care medicine are crucial for formulating and implementing individualized treatment plans (**Table 2**). The rheumatology department is primarily responsible for the immunological assessment of the disease and control of systemic inflammation, using treatment strategies that include corticosteroids, immunosuppressants, and biological agents (75, 76). The respiratory department focuses on monitoring and managing airway function, using pulmonary function tests, dynamic airway imaging (such as dynamic CT), and bronchoscopy for the timely assessment of the airway lesion progression and treatment responses (3, 20). The otolaryngology department plays an important role in diagnosing airway and upper respiratory tract cartilage lesions and in performing airway reconstruction surgeries and related interventions (72, 77). The intensive care department addresses the emergency management of acute respiratory failure and airway obstruction, implementing life-support measures such as mechanical ventilation, tracheostomy, and airway stenting (69, 78).

**Table 2:** Multidisciplinary team (MDT) collaborative framework for managing airway involvement in relapsing polychondritis,.

MDT member specialty	Core role in diagnosis	Core role in treatment	Key collaborative value
Rheumatology	Leads systemic diagnosis and differentials; assesses global inflammatory activity.	Formulates and leads systemic immunosuppressive therapy; manages long-term treatment.	MDT Lead. Integrates inputs to unify local and systemic treatment strategies.
Pulmonary & Critical Care Medicine	Assesses airway structure/function (Pulmonary Function Test, CT); provides diagnostic evidence via bronchoscopy and biopsy.	Performs bronchoscopic interventions; manages respiratory failure and critical care support	Core procedural and acute care platform. Provides key interventions and interfaces with ICU.
Otolaryngology (ENT)	Evaluates upper airways (laryngoscopy); diagnoses characteristic signs (e.g., auricular chondritis, subglottic stenosis).	Manages upper airway stenosis (intervention/surgery); performs tracheostomy and laryngotracheal reconstruction.	Upper airway management specialist. Its early diagnosis and intervention are crucial extensions of overall airway care.
Radiology	Provides precise airway imaging (HRCT 3D reconstruction) and systemic inflammation assessment (PET/CT).	Objectively assesses post-treatment airway structural changes and inflammatory response via serial imaging.	Objective assessor. Provides quantitative imaging evidence for treatment decisions and monitoring.
Pathology	Analyzes biopsy specimens to confirm chondritis and exclude other diseases, providing definitive diagnostic evidence.	Provides critical pathological basis for adjusting or intensifying treatment regimens.	Definitive diagnostic arbiter. Provides the pathological gold standard for diagnosis in complex cases.
Intensive Care Unit (ICU)	Rapidly assesses airway and systemic status of patients transferred with acute respiratory failure.	Provides advanced life support (invasive ventilation, ECMO) for critically ill patients, ensuring safety during high-risk procedures.	Life support safeguard. Provides the necessary platform for safe diagnosis and treatment during critical phases.

In addition, regular imaging and functional follow-ups are indispensable components of a comprehensive management plan. Imaging examinations, including high-resolution CT, dynamic expiratory CT, and ultrasound, can evaluate structural changes, degree of narrowing, and calcification of the airway cartilage, assisting in determining disease activity and efficacy (48, 79). Functional tests such as pulmonary function tests can quantify ventilatory dysfunction caused by airway involvement and monitor dynamic changes in the condition (20). To prevent the deterioration of airway lesions and reduce the risk of recurrence, the multidisciplinary team should adjust treatment plans promptly based on these follow-up results (76).

Moreover, due to the long-term burden of the disease and treatment side effects, patients with RP often experience psychological stress and a decline in quality of life. Psychological support and quality of life improvement measures are equally important in multidisciplinary management. Through psychological counseling, rehabilitation training, and social support, patients can alleviate anxiety and depression, enhance treatment adherence, and improve their overall quality of life (80). In summary, the multidisciplinary comprehensive management of airway involvement in RP not only encompasses multiple clinical specialties but also emphasizes continuous monitoring and individualized adjustments while addressing the psychosocial needs of patients, making it an effective approach to optimizing prognosis and enhancing patients’ quality of life.

### 8.3 Special management for pediatric patients

In pediatric patients with RP, airway involvement is characterized by rapid progression and severe conditions, making early and effective airway management crucial for improving prognosis. Airway narrowing in children progresses rapidly, often accompanied by significant respiratory distress and wheezing, which makes early tracheostomy a key measure to alleviate respiratory distress and maintain airway patency. Multiple studies in the literature have reported that pediatric patients with RP often require emergency tracheostomy due to severe airway involvement to avoid life-threatening situations (14, 25). At the same time, minimally invasive intervention techniques for pediatric patients, such as tracheal balloon dilation, T-tube implantation, and the combined use of absorbable corticosteroid-releasing stents can effectively reduce airway narrowing, minimize surgical trauma, and improve quality of life (13, 14). The application of these minimally invasive techniques is particularly important during stable periods, as they can delay the progression of airway narrowing and reduce the risk of secondary surgeries.

The long-term management of tracheostomy tubes is also one of the challenges in treating pediatric RP. Although tracheostomy can quickly relieve airway obstruction, long-term tube placement can easily result in infections, tracheal narrowing, and other complications, thus necessitating standardized tube care and the regular assessment of airway conditions. In addition, immunosuppressive therapy, as a core strategy for controlling the underlying disease, combined with tracheostomy and interventional treatments, helps reduce the frequency of relapses and the occurrence of severe complications. In clinical practice, glucocorticoids combined with methotrexate, tocilizumab, and other biological agents are widely used in pediatric RP, achieving good efficacy (12, 29). In some cases, autologous or allogeneic hematopoietic stem cell transplantation has shown potential as a rescue measure for severe refractory airway lesions, but relevant experience is limited and requires further research (81).

Although the survival rate of pediatric patients with RP has significantly improved in recent years due to advancements in diagnostic technology and treatment methods, with most patients achieving remission and stability of the disease, challenges remain such as difficulties in early diagnosis, lack of standardized treatment protocols, and insufficient long-term management of complications. To prevent relapses and improve quality of life, strengthening long-term follow-up and focusing on changes in airway function and systemic inflammation control are of great significance (12, 14). Future research should conduct more multicenter large-sample prospective studies to clarify the best airway management strategies and immunotherapy regimens, promoting overall prognosis improvement for pediatric patients with RP.

## 9 Conclusion

RP airway involvement has a significant effect on prognosis, characterized by varied clinical manifestations and complex diagnosis. Although current research offers multidimensional diagnostic and therapeutic pathways, continuous improvement is essential for precision medicine. Early identification relies on multimodal diagnostic methods, particularly imaging and bronchoscopy, although discrepancies in standards necessitate flexible multidisciplinary approaches to enhance identification rates and prevent deterioration.

Corticosteroids and traditional immunosuppressants are effective treatments, with biologics showing promise for refractory cases, although long-term data are lacking. Hence, individualized plans must be dynamic. Interventional therapies can improve airway narrowing but carry risks, highlighting the need for standardized procedures and risk assessments.

In patients with RP airway involvement, a multidisciplinary management model is crucial for improving prognosis and quality of life, requiring collaboration among various specialties for holistic care and individualized plans. Patient education and psychological support further enhance compliance and satisfaction. Future efforts should focus on large-sample, multicenter studies to identify the optimal diagnostic and therapeutic pathways while integrating new technologies and biologics for improved management.

In conclusion, the effective diagnosis and treatment of RP airway involvement require multidisciplinary collaboration, individualized strategies, and innovative technologies, with ongoing improvements in standards being vital for enhancing patient survival and quality of life.
